# Creating three-dimensional magnetic functional microdevices via molding-integrated direct laser writing

**DOI:** 10.1038/s41467-022-29645-2

**Published:** 2022-04-19

**Authors:** Zemin Liu, Meng Li, Xiaoguang Dong, Ziyu Ren, Wenqi Hu, Metin Sitti

**Affiliations:** 1 Physical Intelligence Department, Max Planck Institute for Intelligent Systems, Stuttgart, 70569 Stuttgart, Germany; 2 Institute for Biomedical Engineering, ETH Zurich, 8092 Zurich, Switzerland; 3 Department of Mechanical Engineering, Vanderbilt University, Nashville, TN 37235 USA; 4 School of Medicine & College of Engineering, Koç University, 34450 Istanbul, Turkey

**Keywords:** Mechanical engineering, Fluidics

## Abstract

Magnetically driven wireless miniature devices have become promising recently in healthcare, information technology, and many other fields. However, they lack advanced fabrication methods to go down to micrometer length scales with heterogeneous functional materials, complex three-dimensional (3D) geometries, and 3D programmable magnetization profiles. To fill this gap, we propose a molding-integrated direct laser writing-based microfabrication approach in this study and showcase its advanced enabling capabilities with various proof-of-concept functional microdevice prototypes. Unique motions and functionalities, such as metachronal coordinated motion, fluid mixing, function reprogramming, geometrical reconfiguring, multiple degrees-of-freedom rotation, and wireless stiffness tuning are exemplary demonstrations of the versatility of this fabrication method. Such facile fabrication strategy can be applied toward building next-generation smart microsystems in healthcare, robotics, metamaterials, microfluidics, and programmable matter.

## Abstract (web summary)

Magnetic, wireless miniature devices are promising for healthcare, information technology, and many other field but lack advanced fabrication methods for micrometer length scales. Here authors present a molding-integrated direct laser writing fabrication protocol to fabricate 3D micron-scale devices with programmable magnetization and multi-material integration.

## Introduction

Due to their compact size, precise controllability, and high mobility^1–3^, small-scale wireless devices have shown strong potential in bioinspired robotics^2–4^, micron-scale manipulation^5–7^, and biomedical device fields^8–10^. Among various approaches to realize them, magnetically driven small-scale devices have recently shown promise due to their wireless operation, precision, functionality, down-scalability, and fast response^11–15^. To integrate different magnetic materials and realize anisotropic magnetization properties, various fabrication methods have been proposed for millimeter-size magnetic devices using laser cutting^16–19^ or heating^20^, extrusion printing^11^, ultraviolet lithography-based printing^14,21^, and assembly^22^. For magnetic devices at the microscale, two-dimensional (2D) electron-beam and optical lithography methods have been used to encode magnetization profiles in 2D^13,23^, and these devices can morph into certain 3D structures under controlled magnetic fields (**B**). To further realize the fabrication of true 3D micron-scale magnetic devices, a fabrication approach integrating electrodeposition and a single-step molding process was demonstrated^24^. The mechanically interlocking 3D magnetic structures had isotropic magnetization profiles^24^, which limited the shape-change complexity and programmability of the devices. It is still a great challenge to build micron-scale magnetic devices with functional heterogeneous materials, complex 3D geometries, and 3D programmable magnetization profiles simultaneously.

Two-photon polymerization-based direct laser writing (2PP-based DLW) has been a powerful approach to fabricate 3D microscale structures, material architectures, and robots with down to 100 nm resolution^24–29^. Such 3D-printed microdevices have functions that are jointly determined by the geometries of the designed mechanical structures and the material properties of the used ink. Many customized functional 2PP inks have been developed^30^ for applications in electronics^31^, optics^32^, sensing^33^, displaying^34^, actuators^35,36^, and biomedical scenarios^37^. However, magnetic inks that are compatible with 2PP-based DLW usually suffer from poor performance due to their low loading fractions of the magnetic dopants^29^. On the other hand, 2PP-based 3D lithography methods use a tightly focused near-infrared laser beam^38^. Under such intensive laser, non-transparent magnetic materials absorb large optical energy and generate bubbles which can destruct the printed structures. In addition, these non-transparent materials will prevent the laser beam from reaching the desired printing depth, further deteriorating the printing accuracy. Currently, magnetic nanoparticles can only be printed via 2PP-based DLW at low loading fractions (e.g., 1 µg magnetic nanoparticles per milliliter^29^), which limits the overall magnetization magnitude of the printed structures. Therefore, the magnitudes of magnetic torques and gradient forces that can be exerted on these devices are largely restricted, lowering the overall device performance.

**Fig. 1:**
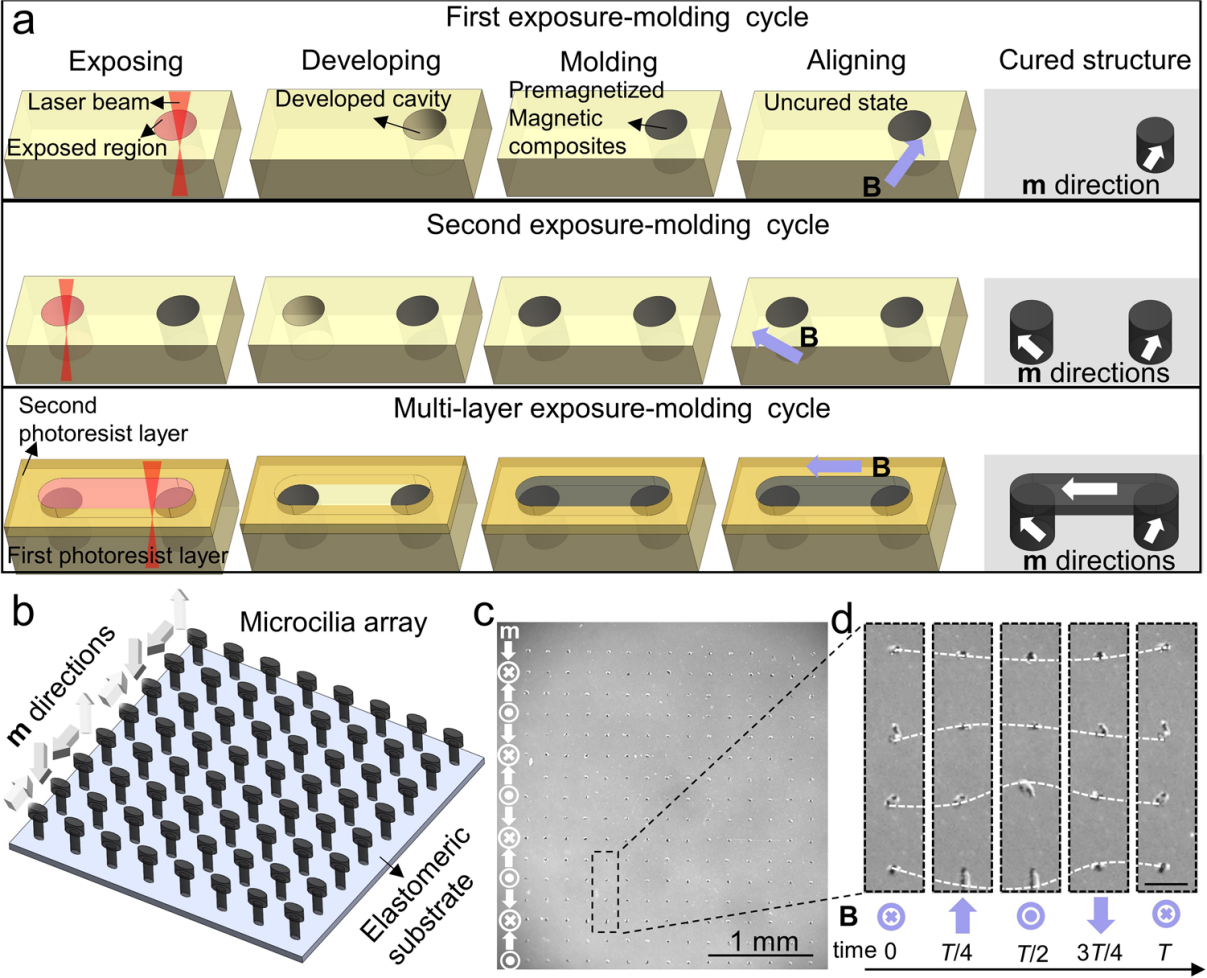
Concept of the multi-step direct laser writing (DLW)-molding process and example showcase of a fabricated magnetic microcilia array. **a** The exposure-molding cycle using 2PP 3D lithography with positive photoresist. Three exemplary molded structures are shown: two standing pillars in the first photoresist layer and a connecting beam in the second photoresist layer. After filling the developed cavity with pre-magnetized magnetic composites in each step, excess material is wiped away from the surface, and an external **B** field will be applied to align the magnetic particles before the composites are cured. **b** Schematic of the fully-soft micro-cilia array with each row encoded with different magnetic moment (**m**) directions which are marked by arrows. **c** Microscopic image of the microcilia array immersed in water. The directions of the arrows mark the **m** directions of all the cilia in that row. The dashed area corresponds to the zoomed-in region. **d** Video (Supplementary Movie 1) frames of the zoomed-in region showing four cilia with different **m** directions bending under an out-of-plane rotating magnetic field. The field is 15 mT and rotates at 0.8 Hz. White dashed lines trace the tip position of each cilium. *T* denotes the period of time of the ciliary beating cycle. Scale bar: 100 µm.

To achieve the goal of fabricating magnetic microdevices with heterogeneous material compositions, complex 3D geometries, and 3D programmable magnetization profiles, we propose a fabrication strategy to mold soft magnetic composites with different properties and integrate the molded parts with 2PP-DLW printed structural architectures. We use 2PP-based lithography to expose positive photoresists and construct complex 3D cavities, which serve as precise negative molds; these 3D structures are molded with magnetic elastomeric composites with high magnetic microparticle concentrations (50 wt%) and their magnetization directions can be arbitrarily aligned in 3D. By repeating this exposure-molding cycle (Fig. 1a), we can integrate multiple materials and program the individual magnetization direction. These magnetic structures are further seamlessly integrated to DLW-printed 3D architectures to form various functional smart microdevices. The unique motions and functionalities enabled by this molding-integrated DLW microfabrication method is showcased by the fabricated magnetic micro-cilia arrays, magnetic micro-rotors, multi-degrees of freedom (multi-DOF) rotary systems, and magnetic micro-mechanical bits (µM-bits).

## Results

### Multi-step micromolding fabrication strategy

We developed a multi-step molding strategy relying on a positive photoresist to build functional 3D microstructures. Although indirect 2PP printing and 3D micromolding using positive photoresist have been demonstrated^24,39^, multi-step molding by repeatedly exposing and developing the same photoresist layer has not yet been reported. This multi-step molding enabled us to sequentially mold different materials and preserve the structures in the previous molded steps. In general, the processing of the positive photoresist involved the steps illustrated in Fig. 1a. First, the viscous positive photoresist was spin-coated on a clean silicon wafer and baked to form a solid layer. Then the femto-second laser beam was focused by an objective lens and scanned to only expose the photoresist at the areas intended to be removed later. Next, the post-exposure bake (PEB) completed the photoreaction and rendered the exposed photoresist soluble in the developer, leaving cavities as molds for filling the desired structures. Pre-magnetized NdFeB microparticle and elastomer composites were filled into the molds; an external **B** (60 mT) field was applied before elastomer curing to orient the magnetized NdFeB particles along the desired direction. This alignment technique is well-established in the literature^11,14^, and we further validated the aligning efficacy by measuring the magnetization of a micro-cilia array with respect to the angular orientation (Supplementary Fig. 1). Up to this point, this was one exposure-molding cycle. The typical life cycle of a photoresist layer normally ends after this one-time molding by dissolving the photoresist with acetone. However, the unexposed part of the photoresist still exhibited photo-reactivity, which enabled us to repeat the whole exposure-molding cycle and align the magnetic materials filled in each step along with different programmed directions. In addition, we could also prepare multiple photoresist layers to mold 3D structures with heterogeneous materials. Finally, after all the molding steps were completed, the photoresist layer was dissolved to retrieve the molded parts.

### Magnetic micro-cilia array with programmable metachronal 2D beating

Researchers have proposed several kinds of artificial cilia that are driven by light^40^, chemical reaction^41^, acoustic wave^42^, thermal and electrostatic actuation^43^, mechanical excitation^44^, pneumatic actuation^45^, and magnetic field^46,47^. Among them, the magnetic ones show better kinematics due to their programmable magnetization profiles. However, the fabrication methods in the literature^46,47^ still lack the capability of encoding phases in cilia arrays at the micrometer scale. Therefore, to showcase the strength of our multi-step exposure and micro-molding process, we fabricated a programmable magnetic micro-cilia array encoded with four magnetization profiles (Fig. 1b). The fabrication steps of this entirely soft microcilia array are shown in Supplementary Fig. 2. Each cilium was 80 µm tall, halved with a 20 µm-wide and 10 µm-thick body with a head of doubled cross-sectional area. The larger heads aimed to accommodate more magnetic particles and thus improved the magnetic torque under external magnetic fields. Here, the average diameter of the magnetic NdFeB microparticles used in this work was five microns. It is possible to downscale the artificial cilia size if we use smaller magnetic particles, but smaller particles usually have a lower magnetization^48^. Although there is an option to increase the actuating magnetic field magnitude to compensate for the lower magnetization, the magnitude of the actuation field should not be larger than the coercivity of the magnetic particles as they would be remagnetized instead of generating a magnetic torque. The limitations on both the allowable maximum external **B** field and decreasing magnetization dictate the possible minimum size of the device. In addition, smaller particles may also increase the viscosity of the composite precursor. However, it can be relieved by a longer waiting time during the vacuum molding process, or by adding an elastomer thinner to decrease the viscosity. The cilia array was molded with the elastomeric magnetic composite (i.e., a silicone rubber elastomer matrix with 50 wt% NdFeB microparticles dispersed inside) in four steps, grouped according to their designed magnetic moment (**m**) directions. The elastomeric composite was magnetized beforehand with a 1 T magnetic field to give NdFeB particles remanent magnetization. After the molds were filled, the elastomeric composite was subjected to a small and uniform magnetic field (60 mT) in the programmed direction during the curing process to align the magnetized NdFeB particles and fix their orientation in the programmed direction. Because of the high coercivity of the NdFeB particles (748 mT), the aligning field was too small to remagnetize the previously cured cilia. Therefore, we were able to build a micro-cilia array with a programmed $\pi$/2 phase difference between each row (Fig. 1c). Here we demonstrated an array size of 15 $\times$ 16, but note that it can be easily scaled up within the printable area of the printing system without extra steps. With a continuously rotating actuation magnetic field (15 mT at 0.8 Hz), each cilium bent, and the array showed a coordinated metachronal wave motion as shown in Fig. 1d and Supplementary Movie 1.

### Reprogrammable and reconfigurable magnetic micro-rotors

**Fig. 2:**
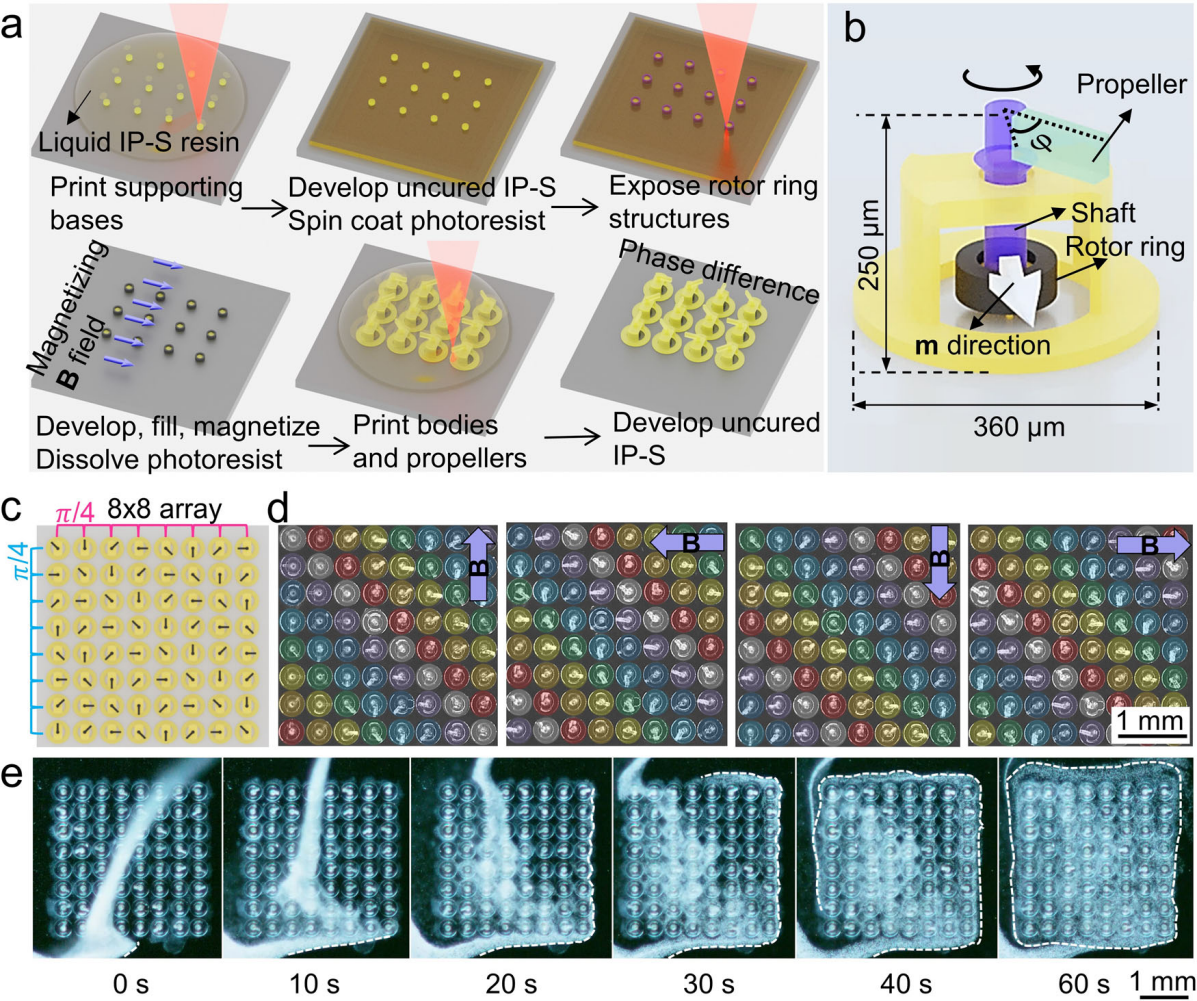
Molding-integrated DLW and micro-rotor array for fabricating programmable micro-actuators. **a** The fabrication process of the micro-rotors. **b** Schematic showing a single micro-rotor. The white arrow marks the magnetic moment (**m**) direction of the rotor ring. The angle between **m** and the propeller is marked as $\varphi$. **c** The arrangement of the propeller orientations in the 8 by 8 micro-rotor array. Neighboring micro-rotors have a phase difference of $\pi$/4. **d** Video (Supplementary Movie 2) frames of the array immersed in water under an in-plane rotating magnetic field. The field is 15 mT and rotates at a frequency of 0.8 Hz. Color code marks the micro-rotors of a certain rotational phase; for example, red denotes a phase of $\pi$/2 and purple does $\pi$. **e** Video (Supplementary Movie 3) frames of the fluidic experiment with the array immersed in water. 2 µm polystyrene beads water suspension is injected at the left bottom corner. The white dashed line marks the contour of the traveling beads. The magnetic field is 40 mT and rotates at a frequency of 5.8 Hz.

Apart from the 2D beating motion, the biological cilia can also generate complex 3D rotation^49^. By using the molding-integrated DLW fabrication approach, we fabricated micro-rotors and achieved phase-coordinated rotating motion at the micrometer scale. The fabrication steps of this molding-integrated DLW are illustrated in Fig. 2a. The micro-rotors had IP-S (a commercial negative 2PP photoresin) rigid bodies with a 360 µm-diameter bottom and a total height of 250 µm (Fig. 2b). The morphology of the micro-rotors was further characterized with scanning electron microscopy (Supplementary Fig. 3a). Each micro-rotor had a NdFeB-elastomeric rotor ring bonded around a pre-printed IP-S supporting base. The base served another purpose; its center could later be connected to the shaft in order to avoid the light path overlapping with the black elastomeric ring. The NdFeB-elastomer composite had a remanence of 39.8 emu g^−1^ and a high coercivity of 748 mT (Supplementary Fig. 4), enabling the rotor rings to rotate with large magnetic torque that could easily overcome friction and fluidic resistance. This large magnetization is otherwise difficult to achieve with DLW-compatible magnetic inks, which are usually doped with magnetic nanoparticles with a weight or volume fraction of only a few percent. We tested the step-out frequency of the micro-rotors inside water; they were able to rotate synchronously with a 10 mT rotating magnetic field up to 12 Hz (Supplementary Fig. 5). All the rotor rings were unidirectionally magnetized by applying a strong magnetic field (1.8 T) after curing the elastomer; therefore, the rotational phase difference between each micro-rotor was solely determined by $\varphi$, the difference between the initial azimuthal angle of the propeller and the **m** direction. As a demonstration, we fabricated an 8 $\times$ 8 array with the neighboring micro-rotors 400 µm apart and programed a $\pi$/4 phase difference between each row and column (Fig. 2c). As seen in Fig. 2d and Supplementary Movie 2, under an in-plane rotating magnetic field, the rotating phases of the propellers propagated with a diagonal frontline. The micro-rotor array could effectively transport and mix fluids using a 40 mT magnetic field rotating at 5.8 Hz as shown in Fig. 2e and Supplementary Movie 3. The marker beads were introduced and quickly transported along the perimeter of the rectangular array. At the same time, a portion of the transported beads infiltrated the space between the rows and columns where the fluids were mixed. The phase profile had unlimited combinations since the propeller of each micro-rotor could be printed to orient along any arbitrary direction. All the supporting bases, rotor rings, bodies, and propellers were printed in single steps, making scaling up easier and faster. Note that this 8 $\times$ 8 micro-rotor array is one specific design. The geometry of a single rotor tip, the spatial distribution of the rotors, and the phase between neighboring rotors can be arbitrarily implemented by our fabrication method.

**Fig. 3:**
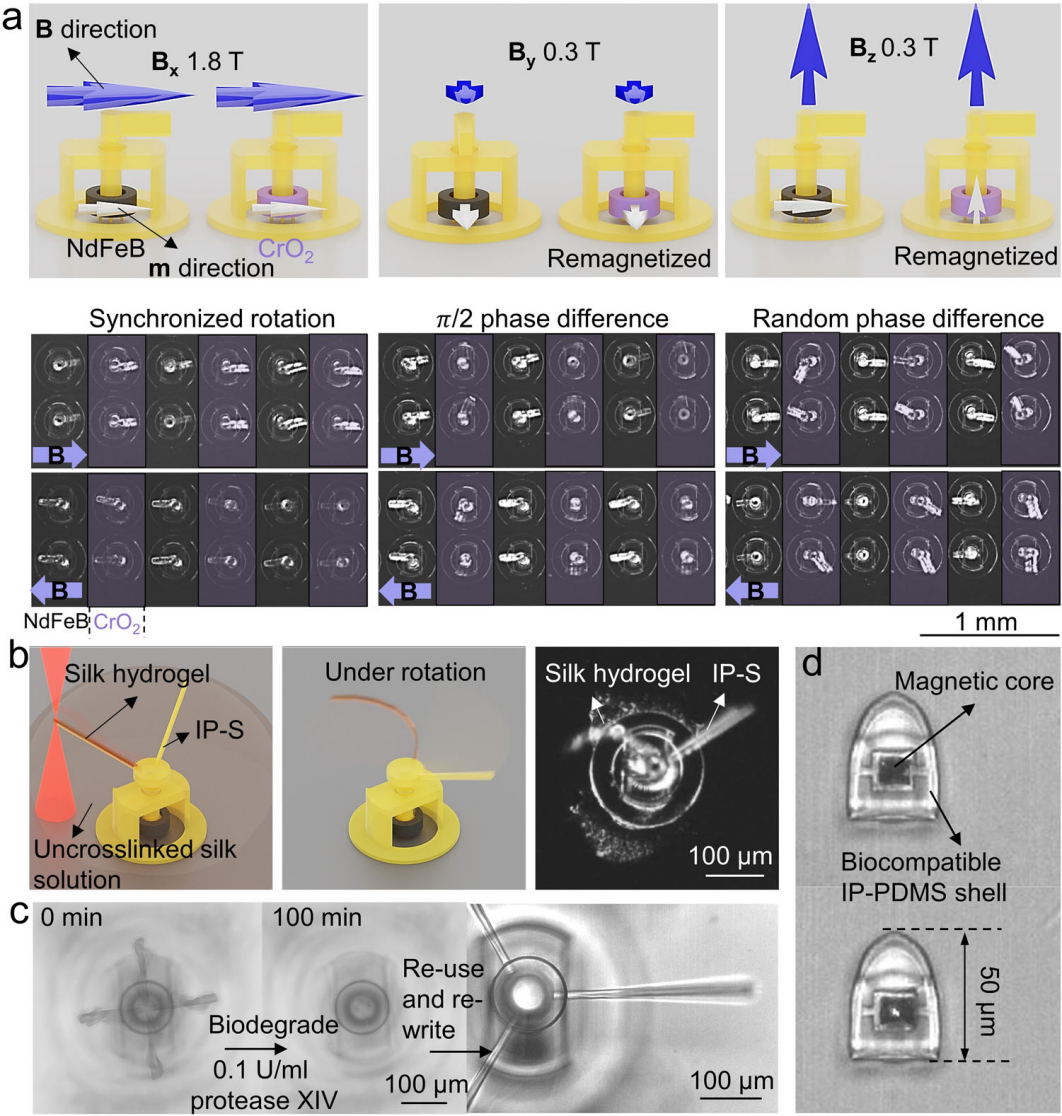
Reprograming, reconfiguring, and passivating the microdevices enabled by DLW and micro-molding with multiple material types. **a** Reprogramming the **m** directions (white arrows) of the micro-rotors by applying magnetic field **B** (blue arrows). The video (Supplementary Movie 4) frames at the bottom correspond to each reprogramming scenario above. The micro-rotors are immersed in water with a 15 mT in-plane rotating magnetic field. The magnetic rings for color shaded rotors are composed of CrO_2_ and the other ones are composed of NdFeB. **b** Schematics showing the DLW process of silk hydrogel and the bending motion under rotation. The video (Supplementary Movie 5) frame shows the spinning motion of the micro-rotor under a 15 mT 0.8 Hz rotating magnetic field in the water. **c** Reconfiguring the geometry by degrading the printed silk “windmill” structure in protease XIV solution at room temperature, then rewriting three beams on the same micro-rotor base. **d** Micro-capsules with fully encapsulated NdFeB-silicone rubber composite core with DLW biocompatible IP-PDMS shell.

In addition, unique features, such as reprogrammability and reconfigurability, are also achievable with this molding-integrated DLW fabrication process. As for reprogrammability, it is a desirable capability for multi-functional and adaptive micromachines—the device can be reprogrammed by external stimuli, preferably without additional structural or material changes. With this fabrication strategy, we can incorporate multiple materials, especially smart materials to endow the microdevices with such advanced capabilities. We demonstrated here reprogrammable magnetic micro-rotors with a low-coercivity ferromagnetic material‒chromium dioxide (CrO_2_). Using a two-step molding process, we could mold the rotor rings in alternate columns with CrO_2_- and NdFeB-elastomeric composites. CrO_2_ and NdFeB are both ferromagnetic materials but with significantly different coercivities, 50 mT and 748 mT, respectively (Supplementary Fig. 4). This coercivity difference enabled one to change the **m** direction of the CrO_2_ rotor rings without affecting the NdFeB ones. As shown in Fig. 3a, all the rotor rings were initially magnetized along the same direction as the propellers by a 1.8 T magnetic field, which was large enough to saturate both types of magnetic materials. Upon a 15 mT rotating magnetic field and immersed in water, all the propellers rotated synchronously as expected (Supplementary Movie 4). Next, we applied an in-plane 300 mT magnetic field that was perpendicular to whichever direction the propellers were resting. At this point of time, all the supporting bases of the micro-rotors were released from the wafer substrate, allowing all rotors to rotate freely. As soon as we applied the remagnetizing field, the NdFeB rotor rings started to rotate and aligned themselves with the field direction. However, a much larger static friction occurred at the contacting surfaces in the air than in the aqueous environment where the rotation experiments were conducted. Due to the weaker remanence of CrO_2_ (18.2 emu g^−1^ comparing with 78.7 emu g^−1^ of NdFeB), the magnetic torque introduced by 300 mT was not enough for the CrO_2_ rotor rings to counter the static friction in the air to start the rotation. All CrO_2_ rotor rings were then subjected to a coercivity-exceeding magnetic field and hence were remagnetized in the direction perpendicular to their last **m** direction. Under the same rotating field, the two types of the micro-rotors now rotated with a ${{{{{\rm{\pi }}}}}}$/2 phase difference. The same remagnetizing field could be applied in any arbitrary direction, including out-of-plane. Since the shafts of the micro-rotors were constrained from free movement other than the rotation motion, the out-of-plane vertically remagnetized CrO_2_ micro-rotors only wobbled around the shaft axis while those comprised of NdFeB remained synchronized with the field rotation.

To realize geometrical reconfiguration, we integrated biodegradable silk hydrogel in the devices. The printed IP-S structures were mechanically robust and did not further react with the printing laser beam, which made it perfect for other DLW-compatible materials to be printed on and attached to as additional mechanical or functional modules. Using the micro-rotor structures as an example, we could directly write the propellers with photo-crosslinkable silk fibroin (SF) hydrogels, which were softer than the crosslinked IP-S by five orders of magnitude of elastic modulus (Fig. 3b and Supplementary Movie 5). The protein-based hydrogel beams were 300 µm long, 10 µm wide, and 20 µm thick. The geometry was well maintained during and after the printing process. Unlike the rigid IP-S propellers, the hydrogel beams resembled the biological cilia and were more capable of complex 3D movements. More interestingly, the printed SF hydrogels could be fully biodegraded in a protease solution under room temperature, as shown in Fig. 3c for a “windmill” structure. The degradation process was faster with higher protease concentration (within 100 min for 0.1 U ml^−1^ and 17 min for 0.5 U ml^−1^) (Supplementary Fig. 6). When necessary, such as being used as sacrificial structures to fix suspending parts temporarily, the printed hydrogels could be quickly removed with a quick rinse in a protease solution. The IP-S structures and the elastomeric rotor rings remained intact during the degradation thus allowing them to be reused and reconfigured. As an example, the windmill structures were degraded, and three new beams were printed and attached to the same micro-rotor shaft (Fig. 3c).

Commonly used strong magnetic materials for sputtering and forming composites (such as nickel, cobalt, and NdFeB) can suffer from undesired corrosion when they interact with the environment, making them cytotoxic thus not biocompatible. Many passivation methods have been developed and tested, such as sputtering another protective layer like titanium^50^, coating the particles with a layer of silica^51^, or coating the whole magnet with parylene^52^. In molding-integrated DLW, we propose enclosing the reactive parts with a more chemically inert photoresin and thus passivate them. We fabricated magnetic micro-capsules with fully enclosed NdFeB composites with biocompatible and durable IP-PDMS^53^, as shown in Fig. 3d, to prevent potential cytotoxicity and immunogenic reactions from occurring.

### Multi-DOF rotary system

**Fig. 4:**
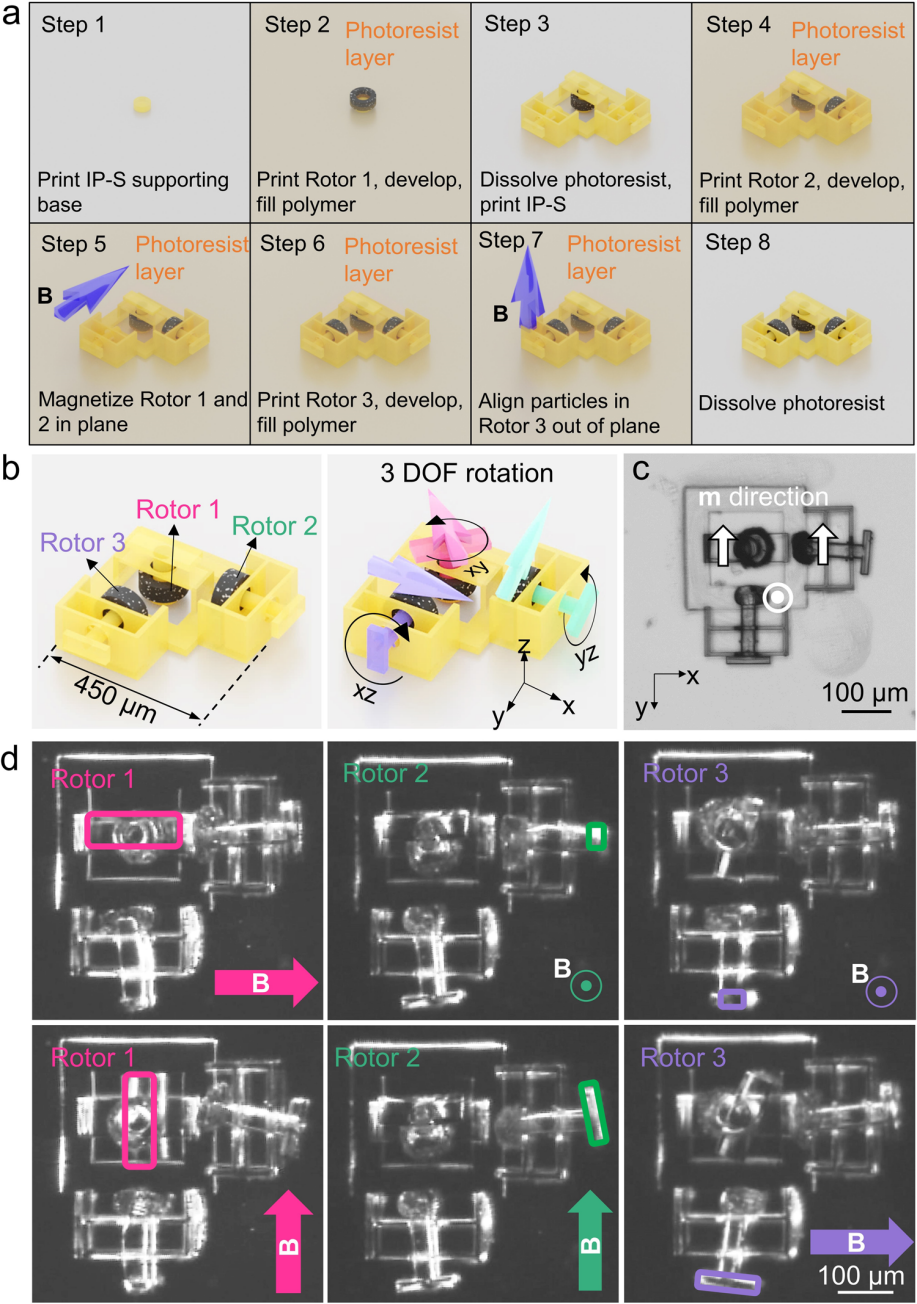
Multi-DOF rotary system enabled by DLW and micro-molding with multi-step casting and dissolvement. **a** The fabrication process of the multi-DOF rotary system. **b** Schematics showing the composition of the system and motions of the three rotor rings. **c** Microscopic image of this device. The magnetization directions of the three rotor rings are marked with white arrows. **d** Video (Supplementary Movie 6) frames showing the three-rotor rings can be separately addressed by applying a rotating magnetic field in different planes. The propellers of each rotor are marked with the corresponding colors. The magnetic field is 15 mT rotating at 0.8 Hz.

It is also possible to make more complex configurations using this fabrication strategy. Using DLW between the molding steps will combine the rigid mechanical parts and all the functional modules into a single entity. As shown in Fig. 4b, we upgraded the single-axis micro-rotor to a 3-DOF rotary system whose three rotors could be individually controlled to rotate in three orthogonal planes, which could not be achieved by sputtering^54^. The rotor rings were molded in three steps across two photoresist casting and dissolving steps. The fabrication process of this micro-rotary system is illustrated in Fig. 4a. After filling and curing the NdFeB-elastomer for Rotor 1 and Rotor 2, a 1.8 T-magnetic field was applied to magnetize both rotors along the *y*-axis. On the contrary, Rotor 3 was composed of a pre-magnetized elastomeric composite; during curing, a small aligning field in the *z*-direction is applied, as shown in Fig. 4c. Rotor 1, Rotor 2, and Rotor 3 were constrained to rotate freely only in the *xy*-, *yz*-, and *xz*-plane, respectively. When a rotating **B** field (15 mT, 0.8 Hz) was applied in one of the three planes, only the corresponding rotor rotated smoothly, while the other two just wobbled around their individual shafts (Fig. 4d and Supplementary Movie 6). The DLW method is able to print arbitrary structures in 3D space. Therefore, it is possible to build the micro-rotors in arbitrarily-oriented planes other than the orthogonal planes and to realize multi-DOF rotation at the micrometer-scale with more complexity.

### Magnetic micro-mechanical bits (µM-bits)

**Fig. 5:**
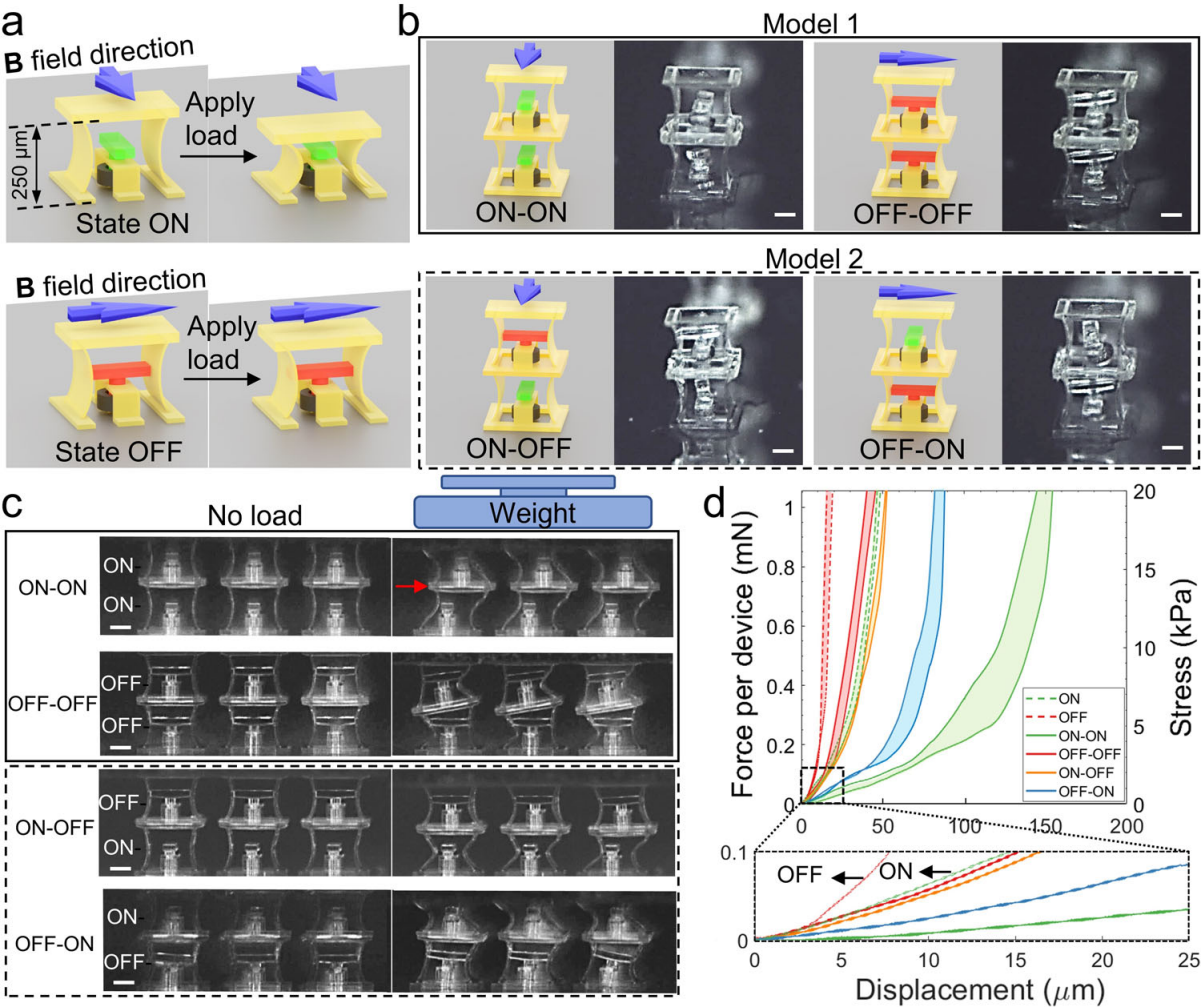
Micro-mechanical-bits (µM-bits) as mechanical memory devices at the microscale enabled by DLW and micro-molding with a multiple-layer process. **a** Schematics showing the working mechanism and design of the µM-bits. Blue arrows mark the **B** field direction needed to align the stopper. **b** double-layer µM-bits configurations and the corresponding microscopic photo of each status. Scale bars: 100 µm. **c** Video (Supplementary Movie 8) frames of the compressing progress of the double-layer µM-bits under each state. Interlayers marked by red arrow have displacement in the shear direction. Scale bars: 100 µm. **d** The force-displacement curves of both single-layer and double-layer µM-bits. The force is measured from nine devices and averaged for plotting. The shaded area denotes the range of measured curves. Low displacement and low force region (dashed area) is zoomed in and the average curve of each sample is plotted for clarity.

So far, all the devices that we have demonstrated were fabricated with a single-layer molding. Next, we will show that it is also possible to build functional multi-layer devices using the proposed molding-integrated DLW strategy. Chen et al. demonstrated mechanical bits (M-bits) using reprogrammable bi-stable metamaterials at the centimeter scale^55^. Each bit could be reversely programmed between two states expressing distinguishable mechanical outputs. As pointed out by a comment article^56^, for practical use, miniaturization and large-scale fabrication of 3D systems of such M-bits is necessary; however, how to achieve that remains unclear. Inspired by that work, we fabricated micro-mechanical bits (µM-bits) with a much-miniaturized double-layer design. The two states, denoted by state ON and state OFF, could be reversely switched between one and the other by changing the orientation of the stopper as shown in Fig. 5a. When the stopper was in the OFF state, under a pressing force, the two side sheets would experience much higher resistance due to the blockage from the stopper; under the OFF state, the µM-bit had a stiffness that was 2.5 times higher than that of the ON state (Table 1). The stopper was printed by DLW and attached to the magnetized rotor ring so that it would rotate and align with the applied magnetic field. Therefore, one could switch between the two states wirelessly using a magnetic field (Supplementary Movie 7).

**Table 1:** Extracted modulus and spring constant of each µM-bits design under both states.

Design	State	Modulus (kPa)	Spring constant (N m^−1^)
1-layer	ON	36.9	7.79
	OFF	90.9	19.2
2-layer model 1	ON-ON	16.3	1.72
	OFF-OFF	75.6	7.98
2-layer model 2	ON-OFF	69.2	7.30
	OFF-ON	35.5	3.75

After completing the first layer, we casted a photoresist layer by a multi-step process of spin-coating and baking to reach a thickness of 300 µm. We molded the rotor rings for the second layer of the µM-bits using the same exposure and development steps as for the first layer (Supplementary Fig. 7). As shown in Fig. 5b, there were two initial configurations possible for the double-layer µM-bits, given that the rotor rings of both layers were magnetized along the same direction. Model 1 denoted the configuration when the two stoppers oriented identically, so they were both in the ON or OFF state. While Model 2 represented those where the two stoppers were printed to be perpendicular to one another. Thus, the two states would be ON-OFF or OFF-ON depending on the direction of the external magnetic field. When the double-layer µM-bits were pressed, the contrast between the ON and OFF layers was obvious (Fig. 5c and Supplementary Movie 8). For example, Model 2, with different top and bottom states, had the majority of the displacement contributed from the ON-state layer since this layer was much easier to deform. The force-displacement results shown in Fig. 5d resonated with the visual cues. The extracted structural modulus and spring constant values are shown in Table 1. Similar to two springs in series, the double-layer Model 1 in ON-ON or OFF-OFF state had a smaller spring constant than the one-layer µM-bits of the same state. From the perspective of output contrast, Model 1 had a larger stiffness difference between the two states, which was 4.6 times than the 1.9 times of Model 2, while one-layer µM-bits had a stiffness difference of 2.5 times between state ON and state OFF. In summary, if one needs stiffer µM-bits, the one-layer design gives a higher spring constant overall; if one cares more about the contrast between the two states, the double-layer Model 1 is a better fit.

To showcase the multi-layer fabrication capability of this method, we fabricated a three-layer µM-bits device (3 × 3 × 3, Supplementary Fig. 8a and Supplementary Movie 9). But practically, when multi-layer µM-bits were pressed, the interlayers of the current design could slide in the shear direction. We could see such phenomena in the two-layer design, especially for state ON-ON (marked by the red arrow in Fig. 5c). When the sliding exceeded a certain extent, the whole structure became instable. This was similar to pressing down a long spring without any non-axial boundaries. This could explain the modulus and spring constant difference between states ON-OFF and OFF-ON. To fix this problem, the current design could be further optimized, for example, making boundaries around each µM-bit to constrain its movement only in the vertical direction.

## Discussion

In this study, we proposed a molding-integrated DLW approach to make magnetic machines with heterogeneous material properties, complex 3D geometries, and 3D programmable magnetization profiles at the micro-scale. The resolution of the mold cavities and DLW geometries could reach sub-micron scale using a 63× objective lens; the only limitation was the size of the magnetic microparticles in the elastomeric composites, which had an average diameter of 5 microns. The molding materials that are compatible with this method are not limited to magnetic elastomer composites. The material space can be extended to other non-DLW-printable functional materials. Exceptions are those whose properties and geometries may be affected by the repeated heating (at ~100 °C) and developing steps or solvents. The molding method is especially suitable for soft materials regardless of their photochemistry and photothermal properties. For more complex designs, a higher step number may be needed, which can be problematic because the photoresist can delaminate from the wafer surface or crack due to repeated heating (because of low solvent content in the photoresist). With careful handling and optimized protocols (see Methods), we were able to mold with the same photoresist layer six times without the photoresist failing. In addition to simple geometries, it is also possible to print molds with complex 3D geometries. As an example, we fabricated a two-layer lattice structure (Supplementary Fig. 8b) using the same magnetic elastomeric composite. Each layer of the structure has 144 unit cells (12 × 12) and each unit cell has a dimension of 40 µm × 40 µm × 40 µm.

The foresight of using smart materials in molding-integrated DLW encourages future work in building physically intelligent microrobots and microsystems^57^. For example, the micro-rotors can be printed in patterns to transport fluids along designed trajectories, or as propellers, they can be integrated with other microstructures to achieve locomotion. The multi-DOF rotary system can be further developed into a mechanical transmissive component with wireless energy input. The future work of µM-bits would aim to develop the capability of reprogramming each bit with either mechanical micromanipulation or using contactless stimuli, such as light^58,59^ and magnetic fields^60^ (see Supplementary Notes). Reprogrammable µM-bits can serve as memory devices using untethered writing and mechanical readings; 3D µM-bits (multi-layers) have multiple stiffnesses corresponding to the combination of the alignment of each stopper, which endows them with higher information density than the current binary memory method. The µM-bits could also be built on a flexible substrate in a large scale and used as haptic interactive components of e-skins. Our strategy of integrating the molding and DLW processes paves the way to the use of a wider selection of functional materials together with the 2PP-DLW technique, enabling unprecedented functional complex micromachine applications in the future.

## Methods

### Materials preparation

Chromium dioxide (CrO_2_) (Sigma-Aldrich Inc) is a metastable strong oxidizer; if the particles are directly mixed with elastomers, curing inhibition occurs^61^. To passivate them, we coated the particles with a layer of silica by the Stöber process, the same method used in literature to coat NdFeB particles^51^. We dispersed 0.4 g CrO_2_ particles in 80 mL ethanol together with 6 mL of 28% ammonia. We kept the suspension vigorously stirred with a homogenizer (Fisherbrand^™^ 150 Watt, Fisher Scientific GmbH). 400 µL tetraethyl orthosilicate in 20 mL ethanol was dropwise added into the stirring CrO_2_ suspension. The mixture was kept on a vortex shaker for 12 h at room temperature, then centrifuged and washed with ethanol three times. After the last wash, the suspension was left in a fume hood to evaporate the solvent in order to retrieve the silica-coated CrO_2_ particles.

NdFeB microparticles (average size 5 µm, MQFP-B, Magnequench) and silicone elastomer kits (Ecoflex^™^ 00-30, Smooth-On Inc; Sylgard^™^ 184, Dow Inc.) were used as received without further modification. Three kinds of magnetic elastomeric composites were prepared: CrO_2_-Ecoflex (weight ratio 1:2), NdFeB-Ecoflex (weight ratio 1:1), NdFeB-PDMS (weight ratio 1:1, PDMS crosslinker ratio 5:1). Because of the low density of CrO_2_, a one-to-two weight ratio was used instead in the CrO_2_-Ecoflex mixture to ensure the flowability of the mixture for molding. The magnetic elastomeric mixtures were prepared by hand-mixing the magnetic particles with the elastomers. For Ecoflex based composites, particles were mixed with part A and part B separately with the same 1:1 weight ratio. To prepare the pre-magnetized magnetic elastomers, the mixture was magnetized using a vibrating sample magnetometer (EZ7, Microsense) with a magnetic field of 1 T.

Regenerated SF was extracted from Bombyx mori silk cocoons using an established protocol^62^. Briefly, *Bombyx mori* silk cocoons were cut into small pieces and were soaked for 30 min in a boiling 0.02 M Na_2_CO_3_ water solution to remove the sericin. The remaining fibroin was then rinsed in deionized water and allowed to dry overnight. The dried fibroin was dissolved in a 9.3 M LiBr solution at 60 °C for 4 h, with a dry-fibroin-weight to LiBr-volume ratio of 1 gram per 4 mL. The dissolved mixture was dialyzed against deionized water for 48 h with eight times of water change. The SF aqueous solution was kept at 4 °C till use. To prepare the photocrosslinkable SF solution, one needs Tris(2, 2′-bipyridyl)dichlororuthenium(II) hexahydrate (Ru(BPY)_3_) and sodium persulfate (SPS). Both reagents were purchased from Sigma-Aldrich without further modification. The formula was modified from literature^63,64^ for 2PP-based DLW. We mixed 100 µL of 5% (w/v) SF solution with 2 µL 0.05 M Ru(BPY)_3_ and 2 µL 0.05 M SPS. The solution was prepared before the DLW step and used fresh since we noticed inconsistent printing results if the solution was prepared the day before.

### Substrate preparation

We used silicon wafers as printing substrates for all devices. Silicon wafers were ultrasonically cleaned in acetone for 5 min and in IPA for another 5 min to clean the surface from organic impurities and dust. The wafers were then baked on a 120 °C-hotplate for 10 min to remove moisture from the surface. This step is important for ensuring a better bonding between the silicon wafer and the photoresist. For DLW, the wafer was used without further treatment. For a better positive photoresist bonding, after the wafers had cooled to room temperature, we spin-coated (3000 rpm, 20 s) a thin layer of adhesion promoter (TI PRIME, Microchemicals GmbH), followed by baking the wafer at 120 °C for 2 min to activate the adhesion-promoting layer. The following photoresist coating was carried out as soon as the wafers are cooled to room temperature.

### Photoresist preparation

The positive-tune photoresist AZ^®^ IPS-6090 (Merck GmbH) was used to fabricate all the molds. The photoresist was spin-coated on the prepared silicon wafer to obtain the desired thickness. The spin-coating speed was determined (90 µm: 800 rpm for 10 s; 60 µm: 1350 rpm for 10 s) from empirical data by measuring the actual thickness at selected spinning speed and fitting the thickness-speed data with an exponential function. The coated-wafers were then baked on a hot plate in two steps. The temperature was first ramped up to 80 °C and was maintained for 3 min, then ramped up to 110 °C and maintained for a period of time depending on the photoresist thickness (90 µm: 9 min; 60 µm: 6 min). The final baking temperature may vary from case to case, depending on the climatic environment (temperature, humidity) in the cleanroom and the shelf-time of the photoresist. During the whole process, one should avoid dramatic temperature change to avoid photoresist cracking due to the difference in heat conductivity and thermal expansion between the silicon wafer and the photoresist layer.

### Printing and developing

A commercial 2PP 3D printing system (Nanoscribe Photonic Professional GT, Nanoscribe GmbH) was used to expose all samples. We used the 25$\times$ NA0.8 immersion objective (Carl Zeiss AG) in all the printing steps. For the positive photoresist exposure, a refractive index matching oil (Immersol 518 f, Carl Zeiss Microscopy GmbH) was applied to the photoresist surface in order to successfully detect the photoresist-wafer interface. Before starting the exposure, the thickness of the photoresist was checked manually by focusing on the photoresist-wafer interface and the oil-photoresist interface. A subtle scratch on the photoresist surface could help with the visualization of finding the oil-photoresist interface. This thickness check aimed to guarantee an accurate placement of the exposed structures in the photoresist layer to be accessible to developers. The exposure parameters were power 20%, speed 13 mm s^−1^, hatching distance 1 µm, and slicing distance 0.3 µm. After printing was complete, the oil was gently removed from the sample surface by wiping with lens tissues. The post-exposure-bake (PEB) is an essential step in completing the photoreaction. We placed the sample on a hot plate, ramped the temperature to 100 °C and maintained it for 100 s. Once the sample was cooled slowly to room temperature, we immersed it in a pool of AZ^®^ 726MIF developer (Merck GmbH) for 3–15 min. The actual developing time was determined by observing the samples under a microscope till all desired features were developed. Finally, we rinsed and soaked the samples in DI water for 2 min and then dried them with compressed air.

At high molding step numbers, the photoresist layer is susceptible to forming cracks; this could result from too low solvent content within the photoresist after multiple PEB heating cycles. One can lower the soft bake temperature or shorten the bake time to ease the cracking problem. Note that a too high solvent content can result in poor photoreactivity, and hence exposed structures may not be developed.

For the DLW of negative photoresin IP-S (Nanoscribe GmbH), we first drop-cast the photoresin on the wafer. When necessary, we degassed the IP-S in a vacuum chamber to remove any trapped bubbles within the printed structures. The printing parameters of IP-S were power 100%, speed 100 mm s^−1^, hatching distance 0.5 µm, and slicing distance 1 µm. The printed IP-S structures were then developed in IPA for 10 min to remove the uncrosslinked resin.

Photocrosslinkable SF solution was drop-cast on the wafer to immerse the fabricated micro-rotor structures. The printing parameters of SF were power 80%, speed 5 mm s^−1^, hatching distance 0.5 µm, and slicing distance 0.5 µm. The printed SF structures were developed in DI water for 5 min to remove the uncrosslinked SF solution.

### Molding

The magnetic elastomeric composites were filled into the photoresist molds by vacuuming for 10 min. At room temperature, the Ecoflex and magnetic particle mixture would start curing once part A and part B were mixed. The curing process was much faster comparing with PDMS, and it became partially cured after just ten minutes. To slow down the curing process and hence give the mixture more time to flow into the molds, we placed ice underneath the wafers during vacuuming. Afterwards, the excess composites were removed by gently wiping the photoresist surface with lens tissues. For 5 min before the heat-assisted curing, a circular Halbach array was employed to apply a 60 mT uniform magnetic field to align the pre-magnetized NdFeB. Then the samples were cured on a 50 °C-hotplate for 15 min (Ecoflex composites) or 1 h (PDMS composites). The Halbach array was still applied during the heating process to ensure that the orientation of the magnetized particles were fixed in the desired direction. For devices on a wafer substrate, we directly dissolved the photoresist after the molding steps were completed. For devices on other substrates, such as silicone rubber, we poured the substrate material on the photoresist surface, degassed it for 5 min, and followed with curing. To dissolve the photoresist, we soaked the wafer in acetone for about 2 min and carefully rinsed the sample with IPA afterwards.

### Design criteria

The molding method is especially suitable for soft materials regardless of their photochemistry and photothermal properties. But for more complex and intricate 3D structures, DLW can deliver results of better precision with fewer steps and thus a faster process. By combining the two techniques, indirect molding, and direct laser writing, one can complement and compensate for the limitation of each individual technique. When designing a structure and creating a processing order, one needs to consider the following points: (1) the mold should be accessible for the soft materials to fill; (2) avoiding overlapping the light path for printing negative photoresin with the molded materials to avoid bubbles; (3) bonding or mechanically interlocking the molded materials and the DLW structures; (4) carefully selecting the molded materials so that their geometries and functions will not be affected by the DLW photoresins or solvents (such as IPA used to develop un-crosslinked negative photoresin or acetone used to dissolve the positive photoresist).

### Fabrication steps of micro-rotors

The molding-integrated DLW method is illustrated in Fig. 2a and details are as follows.

Step 1: Print the supporting bases (diameter: 80 µm, height: 40 µm) using IP-S photoresist on a cleaned silicon wafer and develop in IPA for 10 min. These pillars function as anchors for the non-transparent magnetic rotor rings in the subsequent process.

Step 2: Cast enough amount of AZ-IPS 6090 positive photoresist on the wafer to cover the printed bases and vacuum for 1 min to remove any bubbles. Spin coat (1350 rpm for 10 s) and soft bake (80 °C for 3 min then ramped up to 110 °C and maintained for 6 min) the positive photoresist to obtain a 60 µm-thick photoresist layer.

Step 3: Expose the rotor-ring geometries (outer diameter 120 µm, inner diameter 80 µm, height 45 µm) and follow with PEB and developing steps to obtain mold cavities.

Step 4: Fill unmagnetized NdFeB-PDMS mixtures in the molds by vacuuming. Cure the mixture and magnetize all the rotor rings with an in-plane 1.8 T magnetic field. Dissolve the positive photoresist with acetone and rinse with IPA.

Step 5: Drop-cast negative photoresist IP-S to cover the existing structures and degas it for about 30 min to remove all the bubbles. Print the micro-rotor bodies, shafts, propellers in a single step. The shafts were directly printed onto the supporting bases to bring everything together as an entity. The phase difference was encoded in this printing step by programming the angles of the propellers in the 3D model.

Step 6: Develop the printed structures in IPA for 10 min and then place the wafer in an ultrasonic bath for 30 s to release the supporting bases from the wafer. Since the supporting bases only contacted the wafer via four small pillars (each is 4 µm in diameter), the contact area was much smaller than the one between the body and the substrate. The brief sonication did not release the micro-rotor bodies from the wafer but only released the supporting bases to enable free rotation of the micro-rotors.

### Fabrication steps of multi-DOF rotary systems

The fabrication steps of the multi-DOF rotary device are illustrated in Fig. 4a, and details are as follows.

Step 1: Print the supporting bases for Rotor 1 (diameter 80 µm, height 50 µm) using IP-S.

Step 2: Prepare a layer of 60 µm thick AZ-IPS 6090 photoresist (spin coating speed:1350 rpm for 10 s, soft bake: 80 °C for 3 min, then ramp up to 110 °C for 6 min). Expose the Rotor 1 structures (power: 20%, speed: 13 mm s-1) and obtain the mold cavities after PEB (100 °C for 100 s) and developing (4 min). Fill the mold with unmagnetized NdFeB Ecoflex and cure it for 15 min under 50 °C.

Step 3: Dissolve the photoresist layer with acetone after the elastomeric composite is cured. Print the rest of the mechanical parts using IP-S. The shaft of Rotor 1 can be precisely aligned with the printed supporting base by moving the stage. Connect the shaft with the base by printing with a proper z-overlap.

Step 4: Prepare a layer of 150 µm thick AZ-IPS 6090 photoresist. Repeat the casting, vacuuming (1 min), spin-coating (1350 rpm for 10 s), soft baking (80 °C for 3 min then ramp up to 110 °C for 6 min) process for two times to achieve a layer thickness of 150 µm. Expose the Rotor 2 structures (power: 20%, speed: 13 mm s-1) and obtain the cavities after PEB (100 °C for 100 s) and developing (4 min). Fill the mold with unmagnetized NdFeB-Ecoflex and cure it for 15 min under 50 °C.

Step 5: Magnetize the Rotor 1 and Rotor 2 along y-direction using a 1.8 T uniform magnetic field.

Step 6: Repeat the same step for Rotor 3 as for Rotor 2, except for fill the mold with pre-magnetized NdFeB-Ecoflex composite.

Step 7: Align the magnetic particles by a uniform **B** field in the z-direction, cure the composites for 15 min under 50 °C.

Step 8: Dissolve the photoresist with acetone and place the sample in an ultrasonic bath for 30 s to release the supporting bases from the wafer substrate.

### Setups for actuation experiments

As shown in Supplementary Fig. 9, a DC motor and a Halbach array were used to generate a uniform rotating magnetic field. The motor was controlled by an Arduino board (Arduino Uno, Arduino.cc). The Halbach array had 12 cubic ferromagnets (5 mm × 5 mm × 5 mm, Supermagnet.de). The magnet array was placed 15 mm underneath the sample, which generated a magnetic field strength of 15 mT at the sample plane. At this distance, the magnetic field was uniform in the *xy*-plane within a circular area of ~7 mm diameter which was large enough to cover our samples. All actuation experiments were conducted with microdevices immersed in DI water. The videos were recorded under a stereomicroscope (Stemi 508, Carl Zeiss AG) using a digital camera (GS3-U3-41C6C-C, Point Gray Research Inc.). The micro-rotor array was actuated using a similar setup but a larger Halbach array which gave a 40 mT uniform field at a distance of 5 mm. The motion of the propellers and the fluorescence tracing beads (0.5 µm diameter, Cospheric Inc.) was recorded using a high-speed camera (Phantom MicroLab 140, Vision Research, Ametek Inc.).

### Mechanical measurements of µM-bits

Six samples were prepared for force-displacement measurements, including single-layer state ON and state OFF, double-layer state ON-ON, OFF-OFF, ON-OFF, and OFF-ON. Each sample comprises of a 3$\times$3 array of µM-bits. The measurements were conducted using a house-built setup^65^ with a 25 mN load cell and a high-precision moving stage in the *z*-direction. The wafer was placed on the *xy*-stage directly underneath the load cell. A glass slide approached the µM-bits array at a speed of 1 µm s^−1^ to apply a downward force and retracted at a speed of 50 µm s^−1^ once the 15 mN-setpoint had been achieved. The force and displacement data were recorded during the whole process. After each measurement, the sample was taken off the stage, turned for 90°, re-placed and re-measured for a total of three times. Each layer had a height of 250 µm and a contacting area of 330 µm $\times$16  µm. The modulus and spring constant values were extracted by linear fitting the initial slop of the average force-displacement curve of each sample.

## Supplementary information

Supplementary Information

Description of Additional Supplementary Information

Supplementary Movie 1

Supplementary Movie 2

Supplementary Movie 3

Supplementary Movie 4

Supplementary Movie 5

Supplementary Movie 6

Supplementary Movie 7

Supplementary Movie 8

Supplementary Movie 9

## Data Availability

All data needed to evaluate the conclusions in the paper are present in the paper and the Supplementary Materials.
