# Supplementary material for: Creating three-dimensional magnetic functional microdevices via molding-integrated direct laser writing
Source: Nat Commun. 2022 Apr 19;13:2016. doi: 10.1038/s41467-022-29645-2 (PMC9019016; doi:10.1038/s41467-022-29645-2)
Supplement: Supplementary file 1 — Supplementary Information [file 41467_2022_29645_MOESM1_ESM.pdf]

Supplementary Information

Creating three-dimensional magnetic functional microdevices via molding-integrated direct laser writing

Zemin Liu[1,2,5], Meng Li[1,5], Xiaoguang Dong[1,4], Ziyu Ren[1,2], Wenqi Hu[1*], Metin Sitti[1,2,3*]

[1] Physical Intelligence Department, Max Planck Institute for Intelligent Systems, Stuttgart, 70569 Stuttgart, Germany

[2] Institute for Biomedical Engineering, ETH Zurich, 8092 Zurich, Switzerland

[3] School of Medicine & College of Engineering, Koç University, 34450 Istanbul, Turkey

[4] Department of Mechanical Engineering, Vanderbilt University, Nashville, TN 37235, USA

[5] These authors contributed equally: Zemin Liu, Meng Li

[*] Correspondance to: wenqi@is.mpg.de, sitti@is.mpg.de

Supplementary Notes

Solution to contamination introduced by multi-step molding

The molded structures via multi-step molding may suffer from contamination, when the geometry has a large surface area exposed at the photoresist surface, which can be covered by following molded materials. But for deep structures with small exposed surface area, such as the microcilia array, it is not a major problem because the covering material is much thinner than the underneath structures thus the effect is negligible. If the designed molding structure is a thin layer with large surface area, we suggest using a slightly thicker photoresist layer and print the structure underneath the photoresist surface. To make the structure accessible to the developer, extra tunnels need to be printed to connect the structure and the photoresist surface. Another solution is to spin-coat a thin layer of photoresist (few microns) after the curing process of each molding step to seal the molded structure and protect it from future contamination.

Potential of reprogramming individual µM-bits

There is another reason why we choose to use CrO_2 besides its low coercivity. Previous work[1-3] has shown that CrO_2 has a low Curie temperature at 398 K and a large wide-spectrum photo-absorbance. These two physical properties of CrO_2 make it easy to be demagnetized and remagnetized upon light illumination. Using this ferromagnetic material equips the micromachines with potentials to be selectively reprogrammed via localized light illumination. Our preliminary results validate the feasibility of localized reprogram of the **m** directions at a micrometer scale. We fabricated microcilia with a 3 µm-thickness and 100 µm-length using the molding method and filled with CrO_2-PDMS. We magnetized all cilia along one direction. And we scanned some of the microcilia with the same laser we use for 2PP 3D lithography

while applying a small (20 mT) realigning **B** field in the opposite direction. We observed opposite motion directions from these microcilia under magnetic fields. However, more technical issues need to be resolved, such as laser transmission for thicker structures, integrating an automatically-changing realigning **B** field with the laser scanning process, among others.

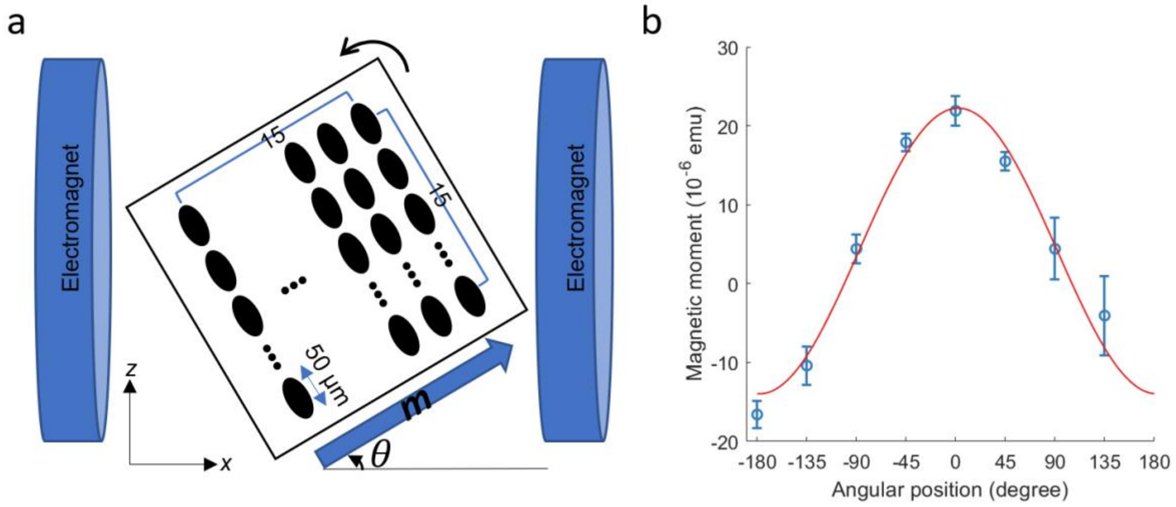

Supplementary Fig. 1 VSM measurement of the characterization sample. a, Schematic showing the measurement setup. The characterization sample has a 15 by 15 array of molded NdFeB-Ecoflex pillars of 40 µm tall, 50 µm long and 25 µm wide. The pre-magnetized particles are aligned along the short axis of the oval cross-section. The sample is rotated in the *xz*-plane and magnetic moment in the x direction is measured. **b**, Magnetic moment measured in related to the rotating angle, θ. Error bars represents standard deviation from 5 readings, and red line is the fitted curve using a cosine function.

Fabrication steps of microcilia array (Supplementary Figure 2)

Step 1: Prepare a layer of 80 µm thick AZ-IPS 6090 photoresist (spin coating speed: 860 rpm for 10 seconds, soft bake: 80 °C for 3 minutes then ramp up to 110 °C for 8 minutes).

Step 2: Expose the cilia structure of the same designed **m** direction (power: 20%, speed: 13 mm s^{-1}) and obtain the cilia-shaped cavities after PEB (100 °C for 100 seconds) and developing (8 minutes).

Step 3: Fill the mold with magnetized 1:1 NdFeB: Ecoflex 00-30 mixture, then align the particles along the desired direction using a uniform external **B** field. Cure the elastomeric mixture on a 50 °C-hotplate for 15 minutes.

Step 4: Repeat step 2 and step 3 three more times to mold all four phases magnetic microcilia.

Step 5: Pour a layer of pristine Ecoflex 00-30 on the surface of the photoresist and cure it for 15 minutes at 50 °C. This elastomeric layer will function as a soft substrate for the microcilia array.

Step 6: Dissolve the photoresist with acetone, rinse with IPA and store the sample in water.

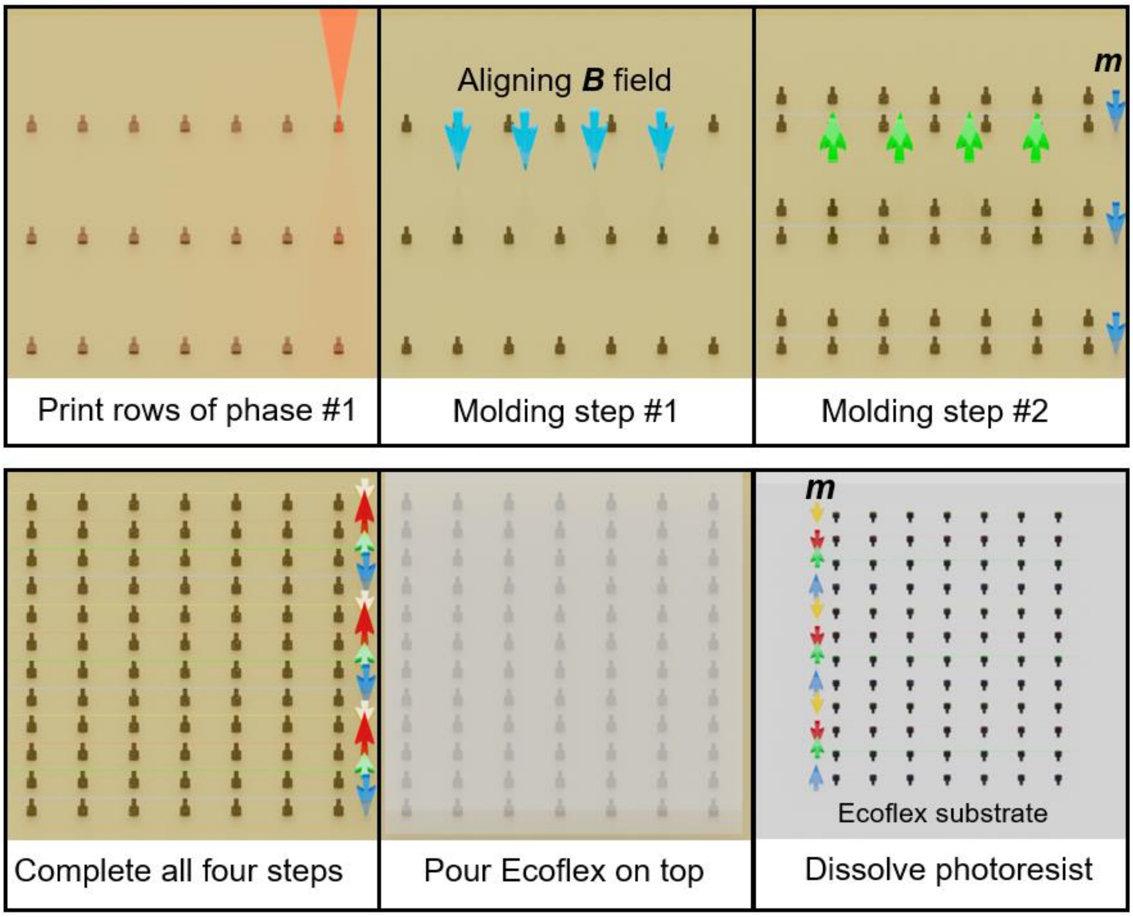

Supplementary Fig. 2 Fabrication steps of the microcilia array with four magnetization phases.

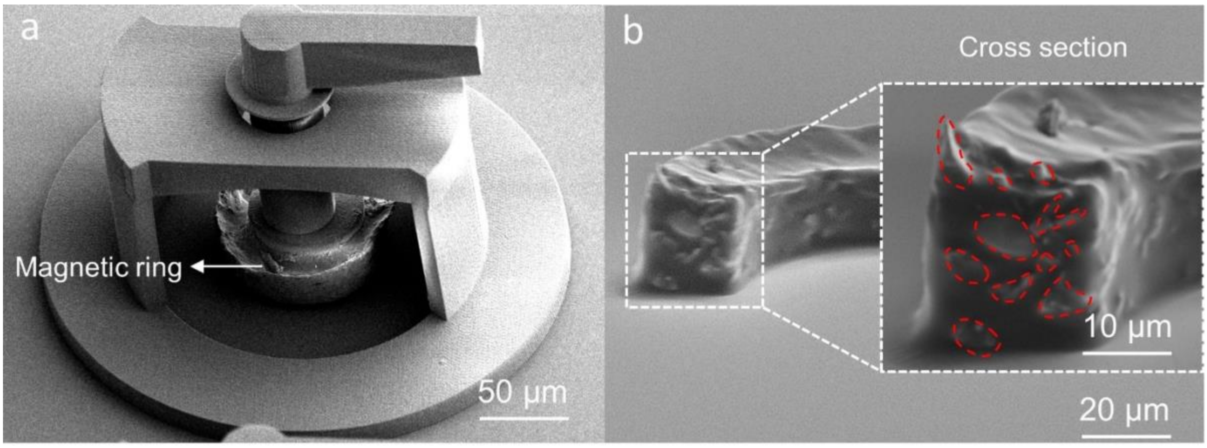

Supplementary Fig. 3 SEM images of a micro rotor and a half magnetic ring. a, A micro rotor. The magnetic ring is marked by a white arrow. **b**, A half magnetic ring. The inset panel is an enlarged cross-section view of this magnetic ring, and the magnetic particles are marked by red dashed line boxes.

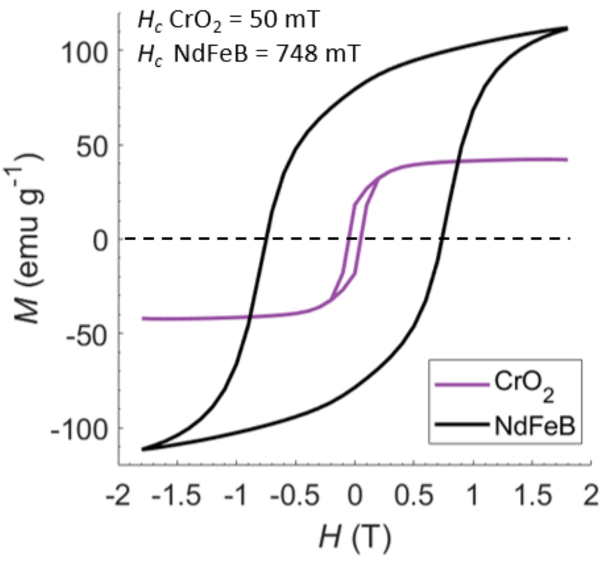

Supplementary Fig. 4 Hysteresis curves of silica-coated CrO_2 particles and NdFeB particles at room temperature.

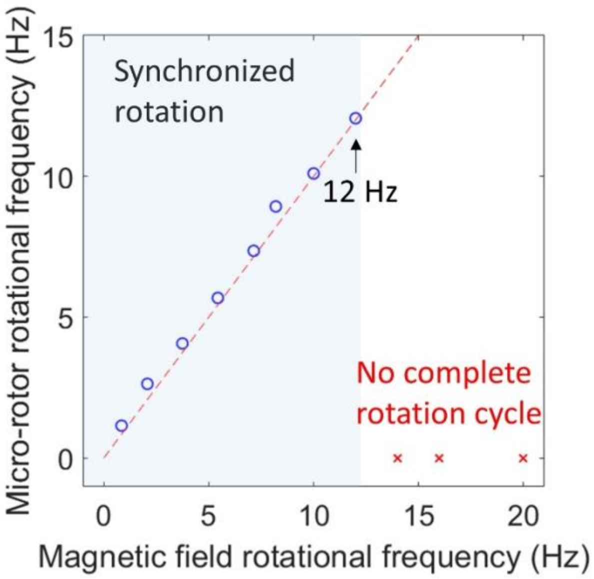

Supplementary Fig. 5 Determination of step-out frequency. Relationship between the micro-rotor rotational frequency and external magnetic field rotational frequency (magnetic field amplitude: 10mT).

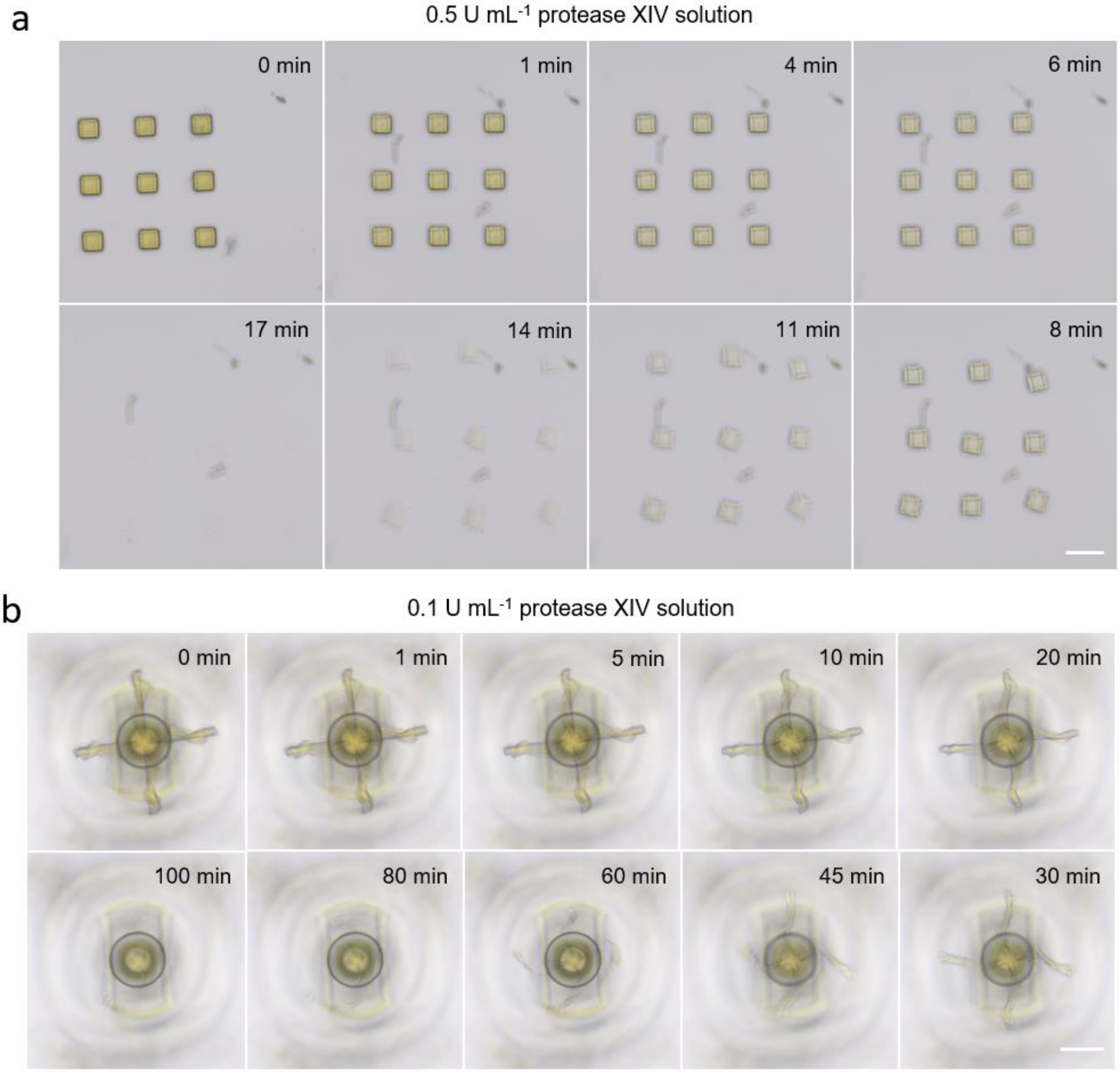

Supplementary Fig. 6 Biodegradation of printed silk fibroin hydrogels. All the silk hydrogels are printed using power 80% and speed 5 mm s^{-1}. The samples are immersed in protease XIV aqueous solution of room temperature of concentration **a**, 0.5 U mL^{-1} and **b**, 0.1 U mL^{-1}. Scale bars: 100 µm.

Fabrication steps of double-layer µM-bits (Supplementary Figure 7)

Step 1: Print the supporting bases for the bottom layer rotor rings (diameter 80 µm, height 50 µm) using IP-S.

Step 2: Prepare a layer of 60 µm thick AZ-IPS 6090 photoresist (spin coating speed :1350 rpm for 10 seconds, soft bake: 80 °C for 3 minutes then ramp up to 110 °C for 6 minutes). Expose the rotor ring structures (power: 20%, speed: 13 mm s^{-1}) and obtain the mold cavities after PEB (100 °C for 100 seconds) and developing (4 minutes). Fill the mold with unmagnetized NdFeB-Ecoflex and cure it for 15 minutes under 50 °C.

Step 3: Dissolve the photoresist layer with acetone after the elastomeric composite is cured. Print the rest of the bottom layer and also the supporting bases for the top rotor rings using IP-S. The second layer supporting bases are loosely connected with the bottom layer by 3D design for easy ultrasonication release. The stopper of the bottom layer can be precisely aligned with the printed supporting base by moving the stage. Connect the stopper with the base by printing with a proper *z*-overlap.

Step 4: Prepare a layer of 300 µm thick AZ-IPS 6090 photoresist. Repeat the casting photoresist, vacuuming (1 minute), spin-coating (600 rpm for 10 seconds), soft baking (80 °C for 3 minutes then ramp up to 110 °C for 6 minutes) process for two times to achieve a layer thickness of 150 µm. Expose the top rotor ring structures (power: 20%, speed: 13 mm s^{-1}) and obtain the molding cavities after PEB (100 °C for 100 seconds) and developing (4 minutes). Fill the mold with unmagnetized NdFeB-Ecoflex and cure it for 15 minutes under 50 °C.

Step 5: Magnetize all the rotor rings in the wafer plane with a 1.8 T-magnetic field.

Step 6: Dissolve the photoresist in acetone. Print the top layer mechanical structures using IP-S. The phase between the bottom and top layers can be realized by changing the angle between the stopper and magnetization. After developing in IPA for 10 minutes, put the sample in an

ultrasonic bath for 30 seconds to release both supporting bases and thus enable the stoppers to rotate freely.

For the fabrication of three-layer µM-bits, repeat the photoresist preparation process in step 4 for two times to prepare a 600 µm thick AZ-IPS 6090 photoresist layer, followed by the same procedure to stack the third layer on the second layer.

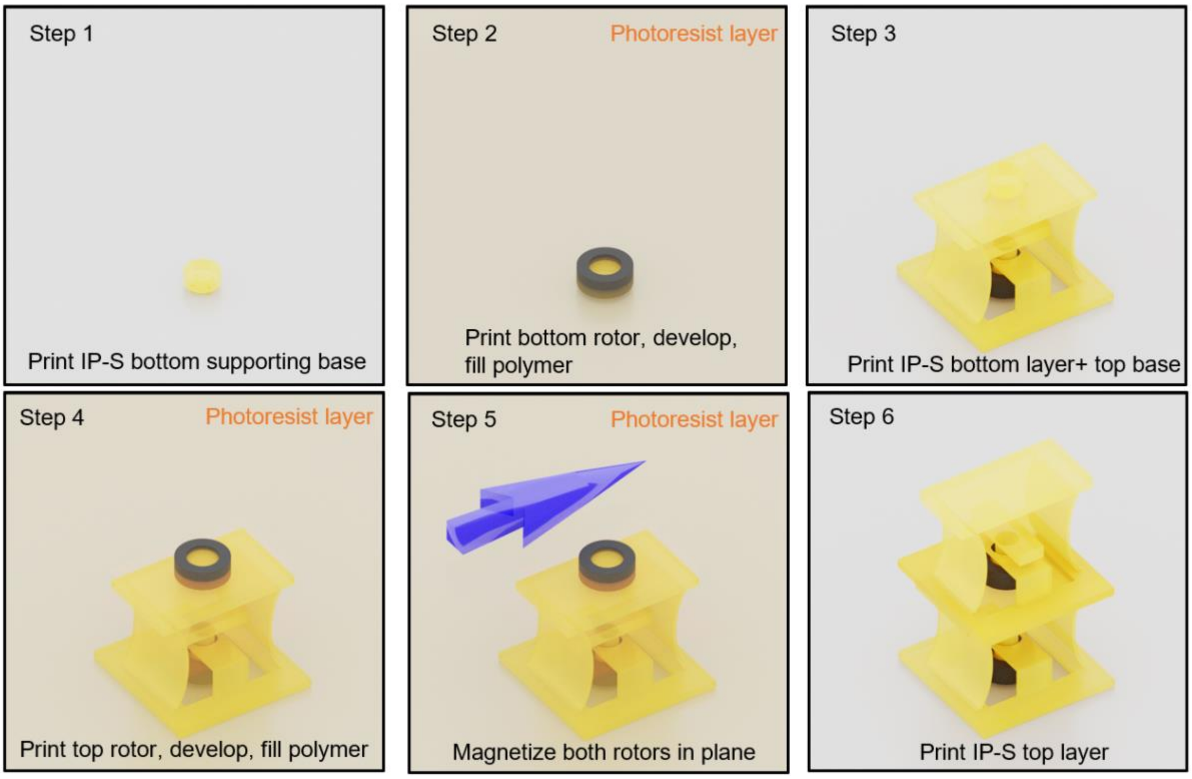

Supplementary Fig. 7 Fabrication steps of the double-layer µM-bits. Blue arrows indicate the applied magnetizing **B** field direction.

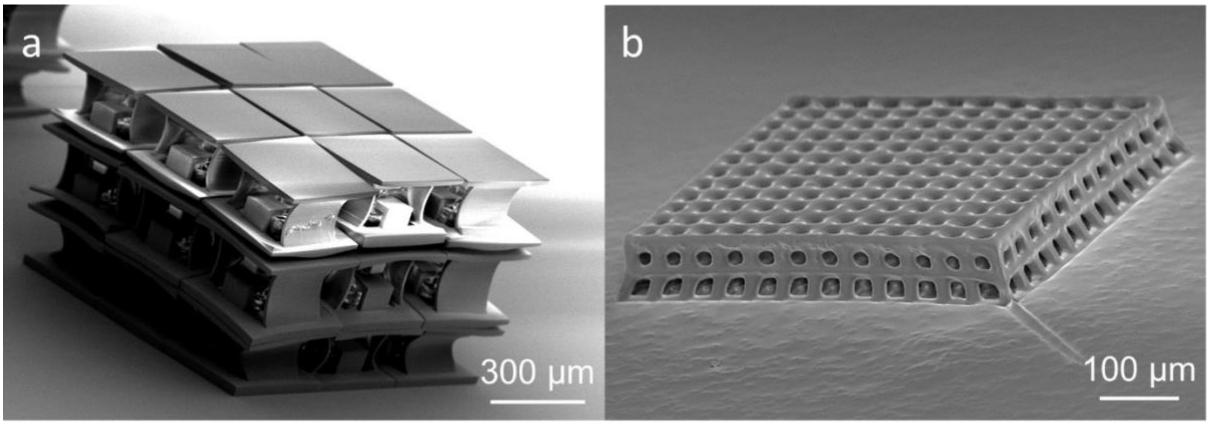

Supplementary Fig. 8 SEM images of three-layer µM-bits and a two-layer lattice structure. a, Three-layer µM-bits. Each layer is composed of nine µM-bits, and each µM-bit has a magnetic ring. **b**, Two-layer magnetic lattice structure. Each layer has 144 unit cells (12×12).

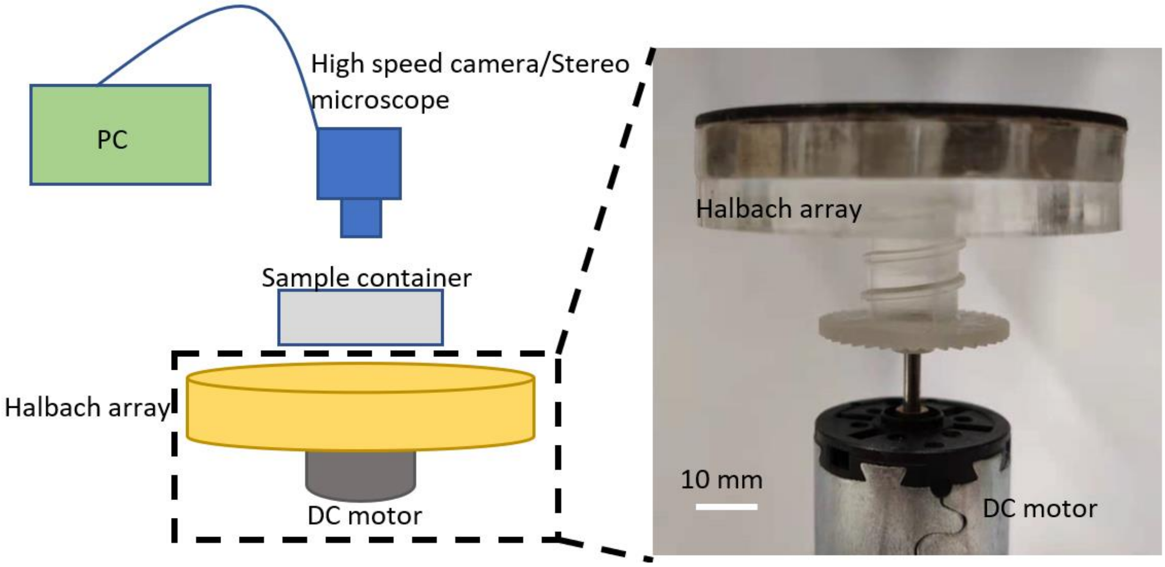

Supplementary Fig. 9 Schematic and photo showing the actuation setup. The rotating Halbach array provides a uniform rotating magnetic fields.

References

1. Li, M. *et al.* Flexible magnetic composites for light-controlled actuation and interfaces. *PNAS* **115,** 8119–8124; 10.1073/pnas.1805832115 (2018).

2. Li, M., Kim, T., Guidetti, G., Wang, Y. & Omenetto, F. G. Optomechanically Actuated Microcilia for Locally Reconfigurable Surfaces. *Advanced Materials* **32,** e2004147; 10.1002/adma.202004147 (2020).

3. Alapan, Y., Karacakol, A. C., Guzelhan, S. N., Isik, I. & Sitti, M. Reprogrammable shape morphing of magnetic soft machines. *Science Advances* **6,** eabc6414; 10.1126/sciadv.abc6414 (2020).
