# Supplementary material for: Creating three-dimensional magnetic functional microdevices via molding-integrated direct laser writing
Source: Nat Commun. 2022 Apr 19;13:2016. doi: 10.1038/s41467-022-29645-2 (PMC9019016; doi:10.1038/s41467-022-29645-2)
Supplement: Supplementary file 2 — Description of Additional Supplementary Information [file 41467_2022_29645_MOESM2_ESM.pdf]

Description of Additional Supplementary Information

Title: Supplementary Movie 1

Description: 2D metachronal beating of microcilia array under a rotating B field.

Title: Supplementary Movie 2

Description: Wave propagation of micro-rotor array under a rotating B field.

Title: Supplementary Movie 3

Description: Fluid transportation and mixing by an 8×8 micro-rotor array

Title: Supplementary Movie 4

Description: Reprogrammability of the multi-material micro-rotors.

Title: Supplementary Movie 5

Description: Motions of the micro-rotors with silk hydrogel propellers under a rotating B field.

Title: Supplementary Movie 6

Description: Motions of the multi-DOF rotary system. Rotors can be individually controlled by applying a corresponding external B field.

Title: Supplementary Movie 7

Description: Alignment of the µM-bits stoppers with a rotating B field.

Title: Supplementary Movie 8

Description: Compressing process of the double-layer µM-bits

Title: Supplementary Movie 9

Description: Alignment of the three-layer µM-bits stoppers with a rotating B field.
